# Ultrasound-guided ilioinguinal-iliohypogastric block (ILIHB) or perifocal wound infiltration (PWI) in children: a prospective randomized comparison of analgesia quality, a pilot study

**DOI:** 10.1186/s12871-020-01170-z

**Published:** 2020-10-03

**Authors:** Bjoern Grosse, Stefan Eberbach, Hans O. Pinnschmidt, Deirdre Vincent, Martin Schmidt-Niemann, Konrad Reinshagen

**Affiliations:** 1 Department of Pediatric Anesthesiology, Altona Children’s Hospital, Bleickenallee 38, 22763 Hamburg, Germany; 2 Center of Experimental Medicine, Institute of Medical Biometry and Epidemiology, University Hospital Hamburg-Eppendorf, Hamburg, Germany; 3 Department of Pediatric Surgery, University Hospital Hamburg-Eppendorf, Hamburg, Germany

**Keywords:** Regional, Ultrasound, opioids, Pain, outpatient, Ambulatory, local, Anesthetics, Drugs, infant, Age

## Abstract

**Background:**

Ilioinguinal-iliohypogastric block (ILIHB) is a well-established procedure for postoperative analgesia after open inguinal surgery in children. This procedure is effective and safe, especially when ultrasound is used. Data availability for comparing ultrasound-guided blocks versus wound infiltration is still weak. The study was designed to determine the efficacy of ultrasound-guided ILIHB (US-ILIHB) on postoperative pain control in pediatric patients following a inguinal daycase surgery, compared with perifocal wound infiltration (PWI) by the surgeon.

**Methods:**

This randomized, double-blinded trail was conducted in pediatric patients aged from 6 months to 4 years. The total number of children included in the study was 103. Patients were allocated at random in two groups by sealed envelopes. The ILIHB group recieved 0,2% ropivacain for US-ILIHB after anesthesia induction. The PWI group recieved 0,2% ropivacain for PWI performed by a surgeon before wound closure. Parameters recorded included the postoperative pain score, pain frequency, time to first analgesics and consumption of analgesics.

*Results:* US-ILIHB significantly reduced the occurrence of pain within the first 24 h after surgery (7.7%, *p* = 0.01). Moreover, the pain-free interval until administration of the first dose of opioids was 21 min longer, on average (*p* = 0.003), following US-ILIHB compared to perifocal wound infiltration. 72% of children who received US-ILIHB did not require additional opioids, as compared to 56% of those who received PWI.

**Conclusion:**

Thus our study demonstrates that US-ILIHB ensures better postoperative analgesia in children and should be prioritized over postoperative PWI.

**Trail registration:**

UIHBOPWIIC, DRKS00020987. Registered 20 March 2020 – Retrospectivley registered.

## Background

The ratio of surgeries performed in an outpatient setting has been increasing rapidly for years and this trend is extend to continue in the future. Open inguinal surgery in children is an outpatient procedure that requires efficient and long-acting analgesia to facilitate early discharge. However, the prevalence of compromising and persistent post-surgery pain in children remains high [1]. Once a child has left the hospital, their parents are tasked with administering medication. Unfortunately, parents often encounter difficulties handling the dosing of painkillers, which results in inadequate or inefficient pain management [2]. Due to the lack of experience and insufficient knowledge of the parents, with respect to pain management, children consequently experience high levels of emotional stress associated with pain. In fact, their understanding of pain has been shown to depend on their psycho-social development stage. Thus, inadequate medical care may result in lasting psychological damage [3]. We must, therefore, focus on the best possible pain treatment. Various randomized studies have demonstrated that local anaesthetic procedures are more effective in children than systemic ones [4, 5]. Local anaesthetic procedures allow for a reduction of the opioid dose which in turn reduces the rate of systemic side effects caused by opioids [6, 7]. A multicentre study by the “Pediatric Regional Anesthesia Network” based on 15,000 cases has demonstrated that the risk of side effects from local anaesthesia in children is low with no observable long-term damage [8]. Hence, the “S3 Guideline on Treatment of Acute Perioperative and Posttraumatic Pain” from 2007, which is currently being revised, recommends using regional anaesthetic procedures, whenever possible, rather than systemic oral painkillers (recommendation grade A) [9]. In fact, the latest Cochrane Review demonstrate that ultrasound-guided regional anaesthetic procedures allow for more targeted blocks using lower doses of local anaesthetics in children, which further reduces the incidence of side effects [10]. Coming to the conclusion that optimal analgesia in surgical interventions can be achieved by means of regional nerve blocks, and the resulting implementation of ultrasound to increase the effectiveness of these nerve blocks, makes the use of ultrasound-assisted nerve blocks virtually indispensable for the prevention of pain in children [11, 12]. The ILIHB to be investigated in this study was first introduced in the 1980’s as an anaesthetic procedure for inguinal surgery in children and did not include ultrasound support. Even though ILIHB is an established regional anaesthesia procedure, data availability for comparing ultrasound-guided blocks versus wound infiltration is still weak due to the lack of evidence [13–18]. Unequivocal data demonstrating that either method provides a high quality of analgesia, in children or in adults, is not yet available making the choice of the right anaesthetic procedure to ensure optimal analgesia difficult.

Our study tested the primary hypothesis that US-ILIHB provides a more adequate analgesia in pediatric inguinal surgery with correspondingly lower pain levels on the pediatric scale of discomfort and pain (KUSS) within 24 h, as compared to surgical perifocal wound infiltration (PWI). Secondarily, we tested the hypothesis that the demand for analgesics after pediatric inguinal surgery is much later in patients of the US-ILIHB group while the amount of painkillers as well as the frequency of their administration is correspondingly lower than in patients of the PWI group.

## Methods

### Approval

The study was reviewed by the ethics committee of the Hamburg Medical Council and approved by the doctoral committee of the University of Hamburg. Parents were informed about the purposes of the study and how their children would be involved at each visit, and their consent was provided in writing. All children were recruited from the pediatric and urological clinic of the Altona Children’s Hospital of the University of Hamburg.

### Power and sample size calculation

The number of cases was calculated using G * Power 3.1. An effect size of 0.6 was derived from previous studies [13, 15]. The alpha was set at 5%. Experience has shown that the drop-out rate is around 10%. Therefore, with a power set to 0.9, the number of 102 cases was calculated, 120 cases are targeted.

### Study population

One hundred sixteen children aged from 6 months to 4 years with a minimum weight of 6 kg; with an American Society of Anesthesiologists Classification (ASA) of I or II and scheduled for a unilateral outpatient inguinal surgery were enrolled in equal randomized (1:1), double-blind, parallel group study conducted in Germany. The initial maximum age of 3 years was extended to 4 years during the study due to the little recruitment number. The following exclusion criteria were applied: mental illness, allergies to relevant drugs, renal insufficiency, coagulation disorders, local infections, emergency procedures, and additional interventions. Demographic data such as gender, age, and weight were collected. Subjects were randomized and allocated to two groups using sealed envelopes including the respective technique (US-ILIHB group, *n* = 53, and PWI group, *n* = 50). After 120 envelopes were numbered 1 to 120, they were filled 1:1 with the technique protocol of the corresponding group. An computer generated simple randomisation allocated the envelops to patients. Only the anaesthesiologist was notified the group and which block technique to use immediately before the induction of anaesthesia when the envelope was opened. After performing the procedure and in accordance with a specified “standardized operation procedure” (SOP), he put the completed technique protocol back into the envelope and sealed it, so that group and corresponding procedure remained hidden from the patient and the personnel performing pain measurements.

### Anatomy

The ilioinguinal and iliohypogastric nerves originate from the spinal cord at the level of L1 and Th12. They cross the inside of the quadratus lumborum muscle to the aponeurosis of the transverse abdominal muscle, which they pierce at the lumbar triangle. Thereafter, they pass between the internal oblique muscle and the transverse abdominal muscle, until entering the internal oblique muscle 1–3 cm medially to the anterior superior iliac spine. In children, on a line between the anterior superior iliac spine and the navel, the ilioinguinal nerve is 9–11 mm away and the iliohypogastric nerve is 13–18 mm away from the anterior superior iliac spine. The ilioinguinal nerve provides sensory innervation to part of the groin: mons pubis and labia or scrotum, with a great range of anatomical variability, especially with respect to the innervation of the labia and scrotum. In 40% of cases, innervation is supplied by the genitofemoral nerve. Iliohypogastric nerve provides sensory innervation to the groin and the skin above mons pubis [19].

### Standardized introduction

Thirty minutes before the induction of anaesthesia, all children were premedicated with midazolam 0.5 mg/kg per os. All patients were intubated and anesthetized according to the SOP as follows: Anaesthesia was induced with sevoflurane via a face mask, 0.3 μg/kg IV sufentanil, and 0.05 mg/kg IV vecuronium. All patients were intubated. Anaesthesia was maintained with 10 mg/kg/h of propofol 1%. IV fluid maintenance therapy was achieved using 1% glucose solution (< 12 months) or 0.9% acetate Ringer’s solution at an infusion rate of 10 ml/kg/h. Children also received 10 mg/kg ibuprofen as at rectal suppository. Lastly 0.1–0.2 μg/kg of sufentanil was administered as a “rescue analgesia” if there were signs of intraoperative pain were detected.

### Block technique

**Fig. 1:**
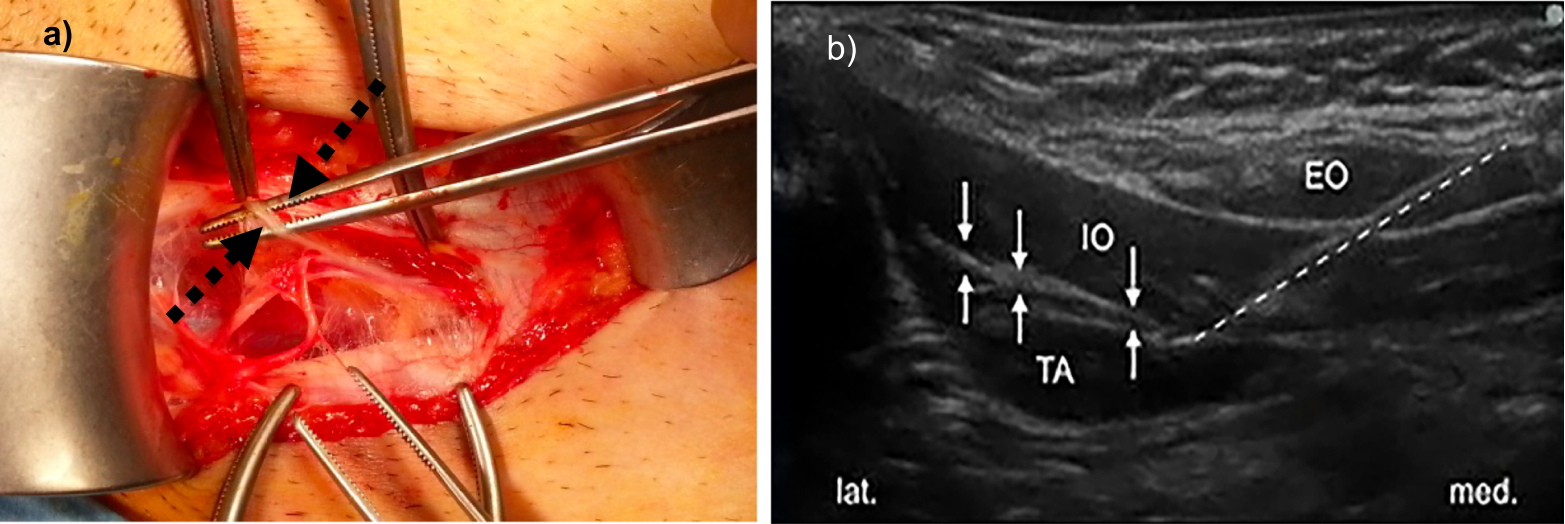
Anatomy: **a** macroscopic: Black arrows = ilioinguinal-iliohypogastric nerves; **b** Ultrasound image: EO = abdominal external oblique muscle, IO = abdominal internal oblique muscle, TA = transverse abdominal muscle; White arrows = Ilioinguinal and iliohypogastric nerves between fasciae, Dotted line = needle in situ; lat.=lateral, med.=medial

As local anaesthetics 0.2 ml/kg of naropin 0,2% was used. This bloc was administered to all patients using sterile conditions and in general anaesthesia. Target structures of the nerve block were the ilioinguinal and iliohypogastric nerves, which run within the fasciae between the oblique abdominal muscles, the internal oblique muscle, and the transverse abdominal muscle (Fig. 1).

**Fig. 2:**
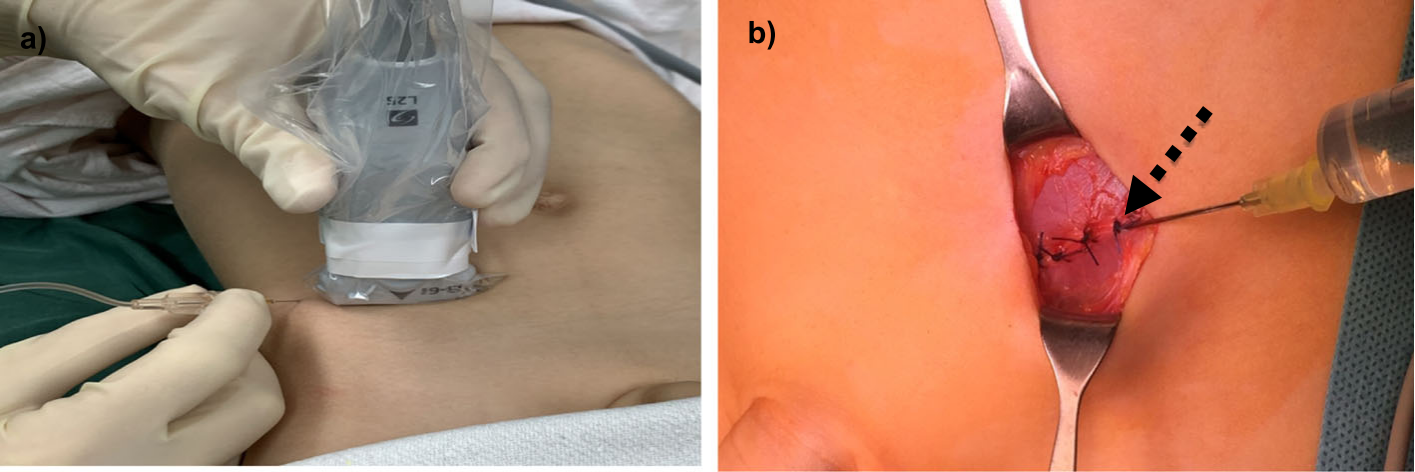
Technique: **a** Ultrasound-guided ilioinguinal-iliohypogastric block; **b** Perifocal wound infiltration after fascial suture, injection through suture gap (arrow)

All patients in the US-ILIHB group were treated by well experienced paediatric anaesthesiologists from the paediatric anaesthesia department of the Altona Children’s Hospital. Immediately after anaesthesia was induced, the main anatomical structures: the external oblique muscle, the internal oblique muscle, and the transverse abdominal muscle were visualized using the ultrasound SonoSite S-Nerv, linear probe (Fig. 1b). A weight-adapted amount of naropin was applied between the internal oblique muscle and the transverse abdominal muscle layers, using a needle guided ultrasound-assisted “in-plane” technique. Prior to injection with naropin a negative aspiration test, via a 25 gauge cannula with 0.5 mm outer diameter, had to be performed (Fig. 2a).

All patients in the *PWI group* were treated by paediatric surgeons from the Department of Paediatric Surgery of the Altona Children’s Hospital. At the end of the surgery, immediately after closing the aponeurosis of external oblique muscle, a negativ aspiration test using a 20 gauge cannula with 0.9 mm outside diameter was administered followed by weight-adapted administration of naropin in macroscopic view through a suture gap (Fig. 2b).

### Pain measurement and postoperative management

Since preverbal children (< 4 years) are not yet able to adequately assess or communicate their level of pain, pain intensity was measured using third-party assessments based on an approved multidimensional pain scale [20, 21], namely the “pediatric scale of discomfort and pain” (KUSS) was used [22]. As primary outcome measure pain measurement datapoints were collected in three instalments within the first 24 h post-surgery. For this purpose, children were transferred to the recovery room, known as the “post-anesthesia care unit (PACU), immediately at the end of anaesthesia at which point the first assessment took place. Trained nurses performed four measurements in 15 minute intervals. Measurements based on KUSS, signs of pain in the categories: crying, facial expression, trunk stance, posture, motor restlessness are each rated with 1-3 points and finally result in a pain score between 0-10, which is intended to reflect pain intensity. Thus a score of 0 means “no pain“ while a score of 10 reflects “maximum pain“. In general, analgesia (0.05 mg/kg piritramide) was provided to children who scored 3 or higher on the KUSS scale until their pain subsided or was at a level of less than 3. Following the stay at PACU patients were transferred from PACU to the discharge station, konwn as the “outpatient surgery ward” (OSW). During the stay at the OSW, the second assessment episode took place, in which nurses performed four measurements at 30 min intervals. After the final examination of the patients and their full recovery, all parents were briefed in detail on the administration of the KUSS scale before the children were discharged. Therefore, the fourth and last assessment episode was performed at home, during which parents performed four measurements at four-hour intervals. In addition to the KUSS scores, complaining of nausea or occurrence of vomiting events, and other abnormalities were queried on the phone the following morning. Secondary outcome measures that were recorded was the time until the first pain medication was administered after surgery (piritramide, ibuprofen, or paracetamol) and lastly, the duration of surgery, and the frequency and total dose of piritramide were also assessed.

### Data analysis

The Statistical data processing was carried out in cooperation with the Institute for Medical Biometry & Epidemiology of the Hamburg Eppendorf University Hospital. The descriptive statistics for continuous variables were based on the mean value and standard deviation per group. Absolute and percentage frequencies per group were determined and presented as categorical variables. Group differences with respect to continuous variables were tested using the Mann–Whitney U test, while group differences with respect to categorical variables were tested with χ^2^– and Fisher’s exact test. The effects of group, time interval, and their interaction with the dichotomous dependent variable were tested using a mixed logistic regression model. A linear mixed model was fit to the continuous dependent variable with the fixed effects of group and point in time, and with the points in time within patients as repeated measures representing repeated measurements. The group-specific course of analgesia administration was analysed and visualized using the Kaplan-Meier method and a comparison of the groups was conducted using logrank tests. The significance level for all tests was set to 0.05 and all statistical tests were set to be bilateral. All analyses were performed using SPSS version 25.0 (IBM, NY, US).

Since the mean values of the collected pain scores of both groups (US-ILIHB and PWI) demonstrated extreme differences among the four measurement episodes, the statistical evaluation was not adequate as planned initially. Pain scores assessed in PACU and OSW were very low in both groups, while pain scores measured at home were relatively high. One reason for this difference was the particularly high occurrence of reports of absolute freedom from pain in the PACU and OSW measurement episodes. In order to be able to present the difference in analgesia between both methods, we planned to analyse the pain scores within the three individual measurement episodes (PACU, OSW, at home), and not on the entire time frame. Thus, in a second step, the data was dichotomized, i.e. divided into values = 0 and > 0. As such, all pain scores > 0 represent pain that occurred at the time of measurement, while pain scores = 0 indicate that there was no occurrence of pain. This allowed us to represent the “relative frequency of occurrence of pain” (rel.freq.), with the resulting differences in rel.freq. Within the episodes (*p* = 0.000) and measurement times (*p* = 0.001), and between groups (*p* = 0.009) being highly significant.

## Results

**Fig. 3:**
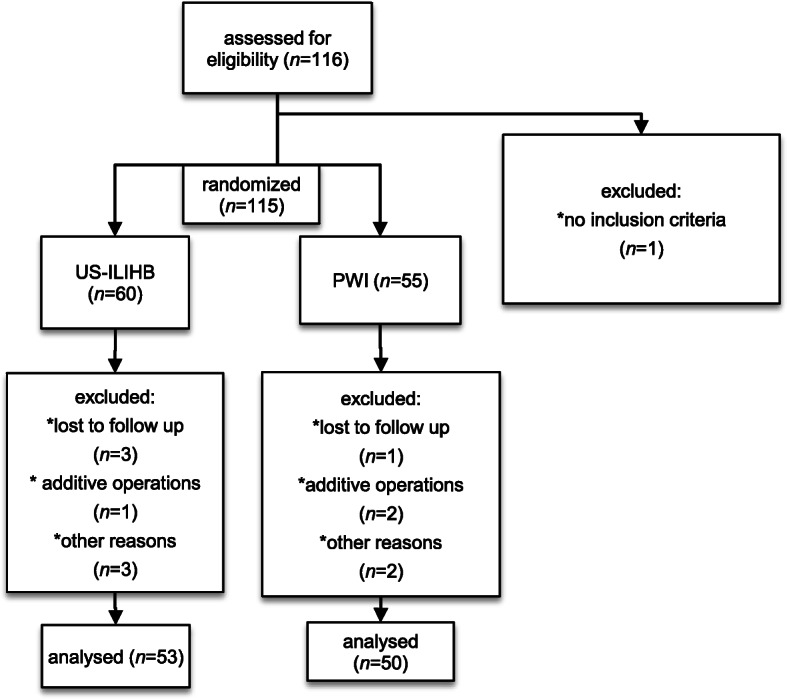
Inclusion procedure flowchart

**Fig. 4:**
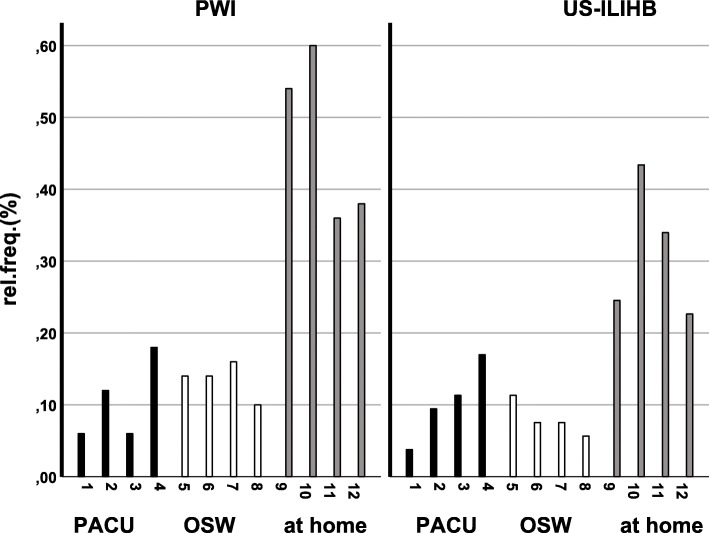
Relative frequency of pain as a function of group and measurement times within the measurement episode (PACU, OSW, at home), 1–12 = measurement times within 24 h

**Fig. 5:**
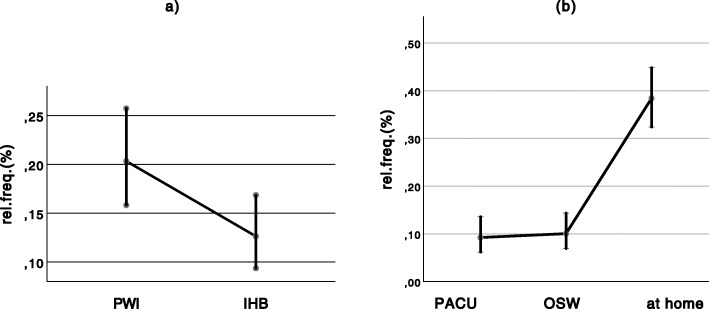
Total relative pain frequency as a function of **a** the group (for all episodes), *p* = 0.01 **b** the measurement episode (for all subjects), *p* = < 0.001

**Fig. 6:**
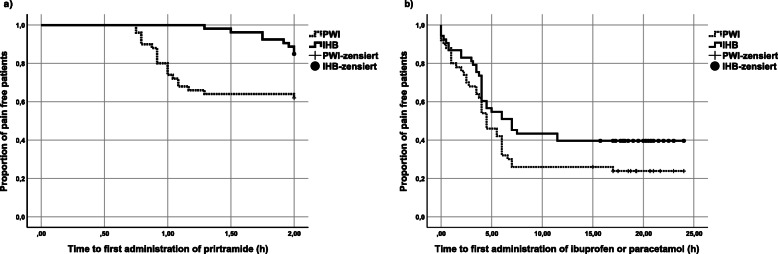
Kaplan-Meier curve; Proportion of pain-free patients up to the first dose of **a** piritramide within the first 2 h after surgery, *p* = 0.003; **b** ibuprofen or paracetamol within the first 15 h at home, *p* = 0.078

**Fig. 7:**
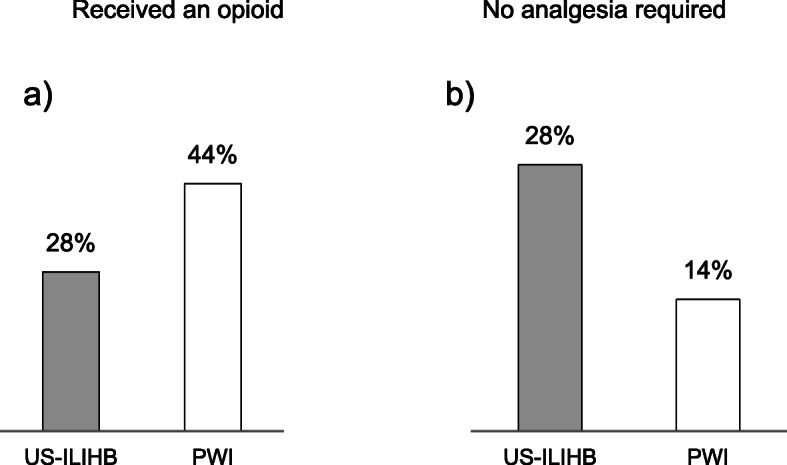
**a** Percentage of children within their group who did not need analgesics; **b** Percentage of children within their group who received an opioid (piritramide). Grey: children in the US-ILIHB group, white: Children in the PWI group

A total of 116 patients were selected for the study, of which, 115 were randomized. Twelve patients were excluded. 53 patients were enrolled and analysed in the US-ILIHB group and 50 patients in the PWI group (see Fig. 3). In the follow up phase we lost contact with four patients, and the study was discontinued for three cases, due to additional interventions having been required. One patient with bronchospasm, during reversal of anaesthesia, and one patient with a known ibuprofen allergy were excluded from the study. Other reasons for exclusion were e.g. gaps in the documentation, or imprecise implementation of the technique due to anatomical challenges. As stated previously, demographics and characteristics of all subjects were recorded (gender, age, weight, ASA, duration of surgery) and shown to have negligible difference on the two groups. The average age in both groups was 2 years, while 83–92% of patient population were boys. The average surgery time was calculated to be 36 min in both groups (Tab. 1). None of the patients treated in the course of this study experienced any complications that could be related to the block technique being investigated. As seen below, Fig. 4 shows a clear trend: Most pain events occurred in the measurement episode at home, with more frequent occurrences of pain in the PWI group than in the US-ILIHB group. Following, results with regards to the respective group (US-ILIHB and PWI) (Fig. 5a) are discussed. The relative frequency of all pain events in the US-ILIHB group was 12.6% (SD = 1.9), whereas in the PWI group it was 20.3% (SD = 2.5), resulting in a difference of 7.7% in favour of the US-ILIHB group (*p* = 0.01). That is, the relative frequency of all pain events in the PWI group is about 50% greater than in the US-ILIHB group. Figure 5b demonstrates the following results obtained with regards to the respective measurement episode (PACU, OSW, at home) and all subjects of both groups, with the frequency of pain being 9.2% (SD = 3.2) in PACU, and 10.0% in OSW (SD = 1.9). Overall, pain was detected most frequently at home with 38.4% (SD = 3.2) (*p* = < 0.001). The time until the administration of piritramide, within the first 2 h after surgery, yielded following results (Fig. 6a): The US-ILIHB group averaged 1.97 h (95% CI 1.93–2.00) until the first piritramide application in comparison to the PWI group, which averaged 1.62 h until the first piritramide application (95% CI 1.48–1.77). This results in a difference of 0.35 h or 21 min of earlier pain treatment in the PWI group (*p* = 0.003). With respect to caregivers’ first administration of either ibuprofen or paracetamol, within the first 15 h of arrival at home, the following results were obtained (Fig. 6b): In the US-ILIHB group, parents administered the first peripheral analgesic after 11.94 h (95% CI 6.07–11.09), whereas in the PWI group required analgesia after 8.58 h (95% CI 9.24–14.64). However, even though the difference is quite large, the findings were not statistically significant (*p* = 0.078) due to large variances in both groups. There were no significant differences regarding the frequency of postoperative nausea and vomiting in both groups within 24 h measurement period: 5 out of 53 versus 5 out of 50 patients reported nausea and vomiting in the US-ILHIB and in the PWI group, respectively. The absolute amounts of administered analgesics and the frequency of analgesia applications did not differ significantly (see Table 1). However, in absolute terms, only 15 children in the US-ILIHB group (28.3%) versus 22 children in the PWI group (44%) received an opioid in PACU. Overall, no analgesics were given to 7 children in the PWI group (14%) versus 15 children (28.3%) in the US-ILIHB group during the 24-h monitoring (Fig. 7).

**Table 1:** Descriptive statistics (upper part), MV = mean value, SD = standard deviation, n = number. Consumption of analgesicis (lower part) for US-ILIHB and PWI group, p = significance

		US-ILIHB (*n* = 53)				PWI (*n* = 50)			
		MW	SD	n	(%)	MW	SD	n	(%)
Sex	F (n/ %)			9	17.0			4	8.0
	M (n/ %)			44	83.0			46	92.0
ASA	I			50	94.3			41	82.0
	II			3	5.7			9	18.0
Age (months)		27.8	14.0	53		26.0	12.9	50	
Body weight (kg)		13.0	3.1	53		12.5	2.8	50	
Duration of surgery (min.)		36.3	14.4	53		36.7	13.4	50	
		MW	SD	n		MW	SD	n	
(n)Piritramide *p* = 0.082		0.4	0.6	53		0.6	0.8	50	
Σ Piritramide (mg) *p* = 0.059		0.2	0.4	53		0.4	0.5	50	
(n)Clonidine *p* = 0.048		0.2	0.6	53		0.4	0.6	50	
Σ Clonidine (μg) *p* = 0.049		3.0	6.5	53		5.9	8.4	50

## Discussion

The primary objective of our research was to demonstrate that targeted ultrasound-guided ILIHB in young children after conventional inguinal surgery, provides better analgesia than surgical infiltration, as this research question has not been answered as to date. In fact, Reid et al. were amongst the first to conducted a study on 49 children in 1987, comparing (1) ILIHB performed using the landmark technique with (2) surgical infiltration, resulting in no significant difference in analgesic effects between both groups [18]. As the block technique was less efficient, since it used anatomical landmarks, it has now been largely replaced by more effective ultrasound technology. Moreover, the number of subjects enrolled in Reid at al.’ s study may have been too low to detect unambiguous differences. Thus, in 1992, Spittal et al. carried out another investigation comparing ILIHB, performed in landmark technique, to surgical infiltration, with a sample of 50 participants. However, this study did not demonstrate any difference either. In 2013, Sahin et al. examined the effectiveness of another abdominal wall block, namely the transverse abdominis plane (TAP) block, that ultimately, achieved a better outcome than the surgical block technique with regards to demonstrate that targeted and ultrasound-guided nerve block achieves better outcomes than a surgical block with regards to (1) time until the first pain medication administration and (2) amount of analgesia used [15]. As Sahin et al.’s results are promising, we decided to investigate the commonly performed ultrasound-guided ilioinguinal-iliohypogastric block. As the ILIHB better targets the anatomical area for inguinal surgical interventions, it has been shown to be more precise, allows for better analgesia, and lower doses of local anaesthetics than the ultrasound-guided TAP method [23, 24]. Our study design enabled us to demonstrate, for the first time, this effect of ultrasound-guided TAP using the example of ultrasound-guided ILIHB. Contrary to the study by Sahin et al., we used a low, uniform dose of 0.2 ml/kg instead of 0.2 and 0.5 ml/kg for both groups in order to more accurately demonstrate the difference in effect between both groups, as overdoses or different dosages between groups can result in possible cofounders.

After many contradictory study findings, the present study provides significant evidence that the relative frequency of pain events is approximately 50% lower in the first 24 h after surgery (12.6% versus 20.4%) when applying US-ILIHB block prior to inguinal interventions in children. In the US-ILIHB group, only every eighth child (6.7 out of 53 children) showed signs of pain, however when surgical infiltration was used, it was every fifth child (10.2 out of 50 children).

We also were able to demonstrate that the frequency of pain events increased the longer the period after block. (Fig. 5b). Thus, as the occurrence of pain in the hospital setting during the first 3 hours was assessed to have been 10%, this was most likely caused by a still intact block. In line with findings, pain increased three times over the next 16 h, as the effect of the block was decreasing.

We also demonstrated that children who received US-ILIHB were longer pain-free, 21 min on average, than children with surgical infiltration, as measured by the time spent until the first opioid administration. Although not statistically significant, the same trend was seen in the time to first administration of peripheral analgesics administered by parents (ibuprofen, paracetamol): In fact, children having received ultrasound block intervention requested an analgesic on average of 3 h later in comparison to their PWI counterparts.

With regards to the consumption of analgesics, the overall volume of consumed opioid analgesics (piritramide) was lower, and they were consumed less frequently following ultrasound block. While these were only minor differences without significance, in context this effect can be interpreted as a trend due to the fact it is also reflected significantly for co-analgesics like clonidine (frequency of clonidine: *p* = 0.048, total clonidine in μg: *p* = 0.049) (Tab. 1, lower part). In general, nearly 50% more children in the PWI group (22/44%) received an opioid in PACU than in the US-ILIHB group (15/28%) (Fig. 7b). However, during 24-h monitoring, no analgesics was given to 7 children (14%) in the PWI group, about half as many as in the US-ILIHB group (15 children, or 30%) (Fig. 7a). Even though differences between the analgesia usage were observed, our study was not set up statistically to answer the question regarding which analgesia was better in terms of quality or intensity, as measured in postoperative pain scores. One reason for this is the high number of pain-free children and this insufficient differentiation of pain scores between the groups. For example, 80% of pain measurements yielded zero in both groups. However, in terms of the quantity and application of analgesics, the cumulation of our results, clearly indicate that the use of US-ILIHB prior to inguinal surgery in young children reduces pain and, consequently, ensures prolonged postoperative freedom from pain with fewer analgesia usages than the surgical PWI at the end of the surgery. Thus, in accordance with almost all investigational criteria, US-ILIHB appears to be significantly superior to PWI, or appears to show a clear trend in that direction.

One reason for our findings is the slow release of anaesthetics, as the preoperative ultrasound-guided application of local anaesthetic in-between fascia of the oblique abdominal wall muscles results in the formation of a deposit. This deposit is then slowly absorbed, thus generating a long-lasting effect (Fig. 1b). Another explanation for US-ILIHB’s superiority over PWI in analgesia properties, is the fact that a surgical injection of analgesia performed before wound closure may be absorbed faster, or may seep through gaps in the fascial suture, resulting in a decreased effectiveness (Fig. 2b). This would also explain the shortened interval until the first administration of analgesics, and the more frequent need for analgesics.

Even though the study’s findings are promising, there are several limitations to address. Firstly, the investigated techniques were not performed by a single person but by several people. However, despite the fact that the group of investigators consisted of a fixed number of specialists within each of departments, all of which have high levels of experience with regional anaesthesia in children, the methods employed were implemented according to a well-established SOP. Therefore, this bias presumably has little effect, given the large number of patients included. Secondly, pain assessments were carried out by nursing staff in the hospital, and by parents at home. Both groups were provided with precise instructions on how to use the same pain scale prior to data collection. The KUSS pain scale is a commonly used and approved scale for the assessment of pain in neonates and small children, however it might still permits a degree of objectivity. Lastly, the temporal offset between applications of local anaesthetics (preoperativ US-ILIHB; postoperativ PWI) could influence pain assessment due to the different residence times of local anaesthetics. However, a clear difference between preoperative or postoperative block in terms of the reduction of pain has not been demonstrated so far [25, 26]. This probably means that this effect is negligible.

## Conclusions

Both methods, ILIHB and PWI, have proven to be effective, with the evidence for better analgesia by one or the other method being thin and ambiguous. Taking into consideration all results presented here, this study demonstrates that the use of pre-operative ultrasound-guided ILIHB could be an improved analgesia method in children (< 4 years old) subjects undergoing open inguinal surgery.

## Data Availability

The datasets during and/or analysed during the current study available from the corresponding author on reasonable request.

## Abbreviations

ASA: American society of anesthesiologists classification
ILIHB: Ilioinguinal-iliohypogastric block
KUSS: Pediatric scale of discomfort and pain
OSW: Outpatient surgery ward
PACU: Post anesthesia care unit
PWI: Surgical perifocal wound infiltration
rel.freq.: Relative frequency of occurence of pain
SOP: Standardized operation procedure
TAP: Transverse abdominis plane block
US-ILIHB: Ultrasound guided ilioinguinal-iliohypogastric block
